# Obesity and COVID-19: Pathophysiological Insights and Pulmonary Complications in a Retrospective Cohort Study

**DOI:** 10.3390/biomedicines13082009

**Published:** 2025-08-18

**Authors:** Cristina Stefania Dumitru, Raul Patrascu, Alexia Manole, Ionut Dragos Capraru, Fira-Mladinescu Corneluta, Felicia Manole, Dorin Novacescu, Flavia Zara

**Affiliations:** 1 Department II of Microscopic Morphology, Discipline of Histology, Victor Babes University of Medicine and Pharmacy Timisoara, E. Murgu Square, No. 2, 300041 Timisoara, Romania; cristina-stefania.dumitru@umft.ro (C.S.D.); novacescu.dorin@umft.ro (D.N.); flavia.zara@umft.ro (F.Z.); 2 Department of Functional Sciences, Victor Babes University of Medicine and Pharmacy, E. Murgu Square, No. 2, 300041 Timisoara, Romania; 3 Faculty of Medicine and Pharmacy, University of Oradea, 410087 Oradea, Romania; manole.alexia@student.uoradea.ro; 4 Department of Epidemiology, Victor Babes University of Medicine and Pharmacy, E. Murgu Sq. Nr. 2, 300041 Timisoara, Romania; ionut.capraru@umft.ro; 5 Hygiene, Department of Microbiology, Center for Study in Preventive Medicine, Victor Babes University of Medicine and Pharmacy, 300041 Timisoara, Romania; fira-mladinescu.corneluta@umft.ro; 6 ENT Department, Faculty of Medicine and Pharmacy, University of Oradea, 410087 Oradea, Romania; fmanole@uoradea.ro

**Keywords:** COVID-19, disease severity, obesity, risk factors, vaccination

## Abstract

**Background/Objectives**: Obesity is a major modifier of COVID-19 outcomes, contributing to increased disease severity and complications. This study aimed to assess the impact of obesity on clinical severity, pulmonary involvement, and in-hospital mortality among COVID-19 patients and to identify independent predictors of severe disease. **Methods:** We conducted a retrospective cohort study of 3005 hospitalized adults with RT-PCR-confirmed COVID-19 between 1 January 2020 and 1 March 2023. Patients were stratified by obesity status (body mass index (BMI) ≥ 30 kg/m^2^). Clinical, comorbidity, imaging, and laboratory data, as well as vaccination status (vaccinated or unvaccinated), were collected. Multivariate regression and gradient boosting models were used to identify predictors of severe outcomes. Effect estimates are expressed as relative risks (RRs) with 95% confidence intervals (CIs). **Results:** Obese patients (*n* = 894) showed significantly higher rates of severe COVID-19 (31.7% vs. 22.4%, *p* < 0.001) and more extensive lung damage (>50% involvement: 27.9% vs. 22.0%, *p* < 0.001), with lower admission SpO_2_ (92.1 ± 4.0% vs. 94.2 ± 3.2%, *p* < 0.001). Hypoxemia (SpO_2_ < 90%) was more frequent in obese individuals. The relative risk (RR) for severe disease was 1.41 (95% CI 1.25–1.60), and for >50% lung involvement, it was 1.27 (95% CI 1.11–1.45). Age > 65 years was the strongest predictor of mortality, particularly in non-obese patients. Gradient boosting models outperformed logistic regression (AUC = 0.92 vs. 0.87). **Conclusions:** Obesity independently predicts severe COVID-19 and pulmonary impairment. These findings support obesity-based risk stratification for clinical management and public health interventions.

## 1. Introduction

The global burden of chronic degenerative diseases (CDDs) has risen dramatically in recent decades, driven in part by the increasing prevalence of obesity [1]. Once considered a simple imbalance of caloric intake and expenditure, obesity is now widely recognized as a complex, multifactorial condition that affects systemic metabolism, immune responses, and susceptibility to acute infections. Its classification as a chronic, noncommunicable disease highlights its long-term impact on health and its pivotal role in the progression of cardiometabolic and respiratory comorbidities [2].

In parallel, the coronavirus disease 2019 (COVID-19) pandemic has provided a striking example of how pre-existing chronic conditions, particularly obesity, can exacerbate the severity of acute viral infections [3]. Numerous studies have identified obesity as a significant risk factor for increased morbidity and mortality among hospitalized patients with COVID-19 infection. Mechanistically, excess adiposity is associated with low-grade chronic inflammation, impaired respiratory mechanics, altered immune function, and metabolic dysregulation—all of which may contribute to worse clinical outcomes in COVID-19. Vaccination against SARS-CoV-2 has significantly altered the clinical trajectory of COVID-19; however, individual metabolic factors, such as obesity, may modulate vaccine response and residual disease severity [4,5]. Given this context, vaccination status represents a critical modifier of COVID-19 outcomes and must be accounted for when evaluating the independent contribution of obesity to disease progression.

Understanding how obesity influences the clinical course of COVID-19 is not only crucial for acute care management but also offers broader insight into the systemic vulnerabilities induced by CDDs [6]. Hospital-based data from diverse populations suggest that obese individuals are more likely to experience severe pulmonary involvement, longer hospitalization, higher rates of intensive care unit (ICU) admission, and elevated mortality compared to their non-obese counterparts. These patterns are particularly concerning given the global rise in obesity rates and the persistent threat posed by emerging viral pathogens [7].

This study aims to evaluate the impact of obesity on the severity and clinical outcomes of COVID-19 in a large cohort of hospitalized patients while explicitly accounting for vaccination status. By analyzing indicators such as pulmonary involvement, disease evolution, the length of hospitalization, and mortality, we seek to clarify the role of obesity as an independent prognostic factor in the context of viral respiratory infections. Furthermore, these findings emphasize the need to integrate nutritional risk assessment and personalized dietary interventions into long-term public health strategies for the prevention and control of chronic degenerative diseases. We hypothesize that obesity independently increases the risk of severe COVID-19 and extensive pulmonary involvement, regardless of age, sex, vaccination status, and baseline oxygen saturation. The primary objective of this study is to evaluate the association between obesity and COVID-19 severity, pulmonary impairment, and in-hospital mortality while identifying independent predictors of severe outcomes using both multivariate logistic regression and machine learning approaches.

## 2. Materials and Methods

### 2.1. Study Design and Population

This retrospective cohort study included adult patients (≥18 years) admitted to the Victor Babeș Clinical Hospital for Infectious Diseases and Pneumophthisiology in Timișoara, Romania, between 1 January 2020 and 1 March 2023. All patients had a confirmed diagnosis of COVID-19 infection via reverse transcription polymerase chain reaction (RT-PCR) at the time of admission. The study cohort comprised a total of 3005 patients with complete clinical documentation and available anthropometric data.

Patients were stratified based on the body mass index (BMI), calculated as weight in kilograms divided by the square of height in meters (kg/m^2^), according to the World Health Organization (WHO) classification: underweight (<18.5 kg/m^2^), normal weight (18.5–24.9 kg/m^2^), overweight (25–29.9 kg/m^2^), and obese (≥30 kg/m^2^) [8]. For the purposes of this analysis, the primary comparison was conducted between obese and non-obese patients.

### 2.2. Data Collection

Data were extracted from electronic medical records and included demographic variables (age, sex, and environment of origin), BMI, vaccination status (the number of doses and time since last dose), and comorbidities (diabetes, cardiovascular diseases, chronic pulmonary disease, renal or hepatic disorders, and neoplasms). The clinical outcomes assessed were as follows:

- The severity of COVID-19 (mild, moderate, or severe), as defined by the WHO criteria [9];
- Pulmonary involvement, based on chest imaging (X-ray or Computed Tomography scan), classified as mild (<25%), moderate (25–50%), or severe (>50%);
- Evolution during hospitalization (recovered, improved, stable, deteriorated, or death);
- ICU (intensive care unit) admission;
- The length of hospital stay (days).

The main variables of interest included obesity status, age, sex, vaccination status, comorbidities, oxygen saturation on admission, and pulmonary involvement.

BMI assessment: Weight and height were measured on admission by trained nursing staff using a calibrated hospital scale and stadiometer, with patients wearing light clothing and no shoes. The body mass index (BMI) was calculated as weight (kg)/height (m^2^). No self-reported anthropometric data were used.

Vaccination status was recorded for all patients and included the number of COVID-19 vaccine doses received and the time elapsed since the last dose. For analysis, patients were categorized as vaccinated (at least one dose) or unvaccinated.

### 2.3. Statistical Analysis

Statistical analysis was performed using MedCalc^®^ version 23.1.7 (MedCalc Software Ltd., Ostend, Belgium) and Python (v3.11.6, scikit-learn library). Descriptive statistics were used to summarize demographic and clinical variables. Continuous variables were assessed for normality using the Shapiro–Wilk test and reported as mean ± standard deviation or median (IQR), depending on distribution. Categorical variables were presented as frequencies and percentages. Group comparisons between obese and non-obese patients were conducted using the chi-squared test for categorical variables and the Mann–Whitney U test for continuous variables. A *p*-value < 0.05 was considered statistically significant.

Although relative risk (RR) is generally more appropriate for cohort designs, including retrospective cohorts, we opted to report the main effect estimates as odds ratios (ORs) in order to maintain consistency across multivariate logistic regression analyses and facilitate comparison with other studies in the literature. This choice was made a priori and applies to all regression-based analyses presented in this manuscript.

To evaluate the independent effect of obesity on clinical outcomes, multivariate logistic regression models were constructed, adjusting for age, sex, vaccination status, and comorbidities. Adjusted odds ratios (aORs) with 95% confidence intervals (CIs) were reported for outcomes including severe COVID-19, pulmonary involvement, and in-hospital mortality. Subgroup analyses were conducted separately for obese and non-obese patients to identify risk factors for mortality.

A gradient boosting model was additionally developed to predict severe COVID-19 and compared with logistic regression. Model performance was evaluated using receiver operating characteristic (ROC) curves and area under the curve (AUC). In addition to the AUC, we reported the sensitivity and specificity at the optimal cut-off point, as determined by the Youden index. Feature importance was analyzed to identify the most influential predictors. Vaccination status was included as a covariate in all multivariable models to control for potential confounding effects.

## 3. Results

### 3.1. Characteristics of the Study Population

Of the 3005 patients included in the study, 894 (29.7%) were categorized as obese (BMI ≥ 30 kg/m^2^), while the remaining 2111 (70.3%) were non-obese. Table 1 summarizes the baseline demographic characteristics of the two groups. The mean age was comparable between obese and non-obese individuals (62.8 ± 15.6 vs. 63.5 ± 14.8 years, *p* = 0.198). The proportion of male patients was slightly lower in the obese group (48.9%) compared to the non-obese group (50.9%), without reaching statistical significance (*p* = 0.337). A significantly higher proportion of patients from urban areas was observed among non-obese individuals (60.2% vs. 56.8%, *p* = 0.0315). Regarding smoking status, 28.1% of the obese patients were current smokers, compared to 27.8% in the non-obese group (*p* = 0.055). Although not statistically significant, this value is close to the conventional significance threshold and may indicate a trend toward a higher proportion of smokers in one of the groups. Regarding vaccination status, 623 patients (20.7%) were vaccinated (at least one dose) and 2382 (79.3%) were unvaccinated.

### 3.2. Association Between Obesity and COVID-19 Severity

**Figure 1:**
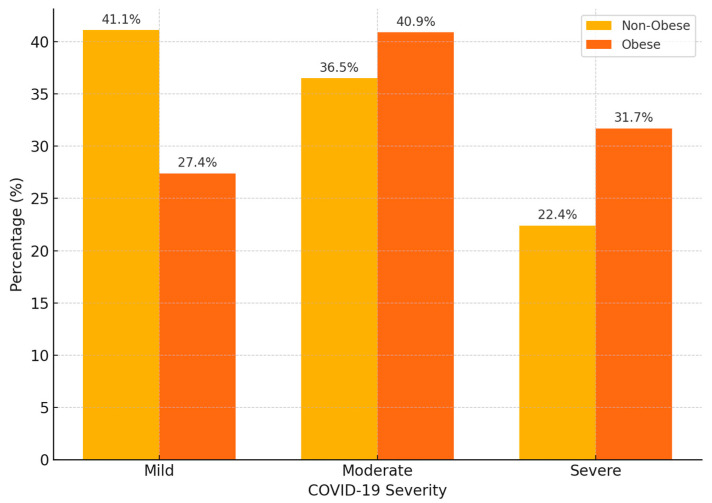
Th distribution of COVID-19 severity by obesity status. The bar chart shows the percentage of mild, moderate, and severe cases among obese and non-obese hospitalized patients.

The severity of COVID-19 varied significantly between obese and non-obese patients, as shown in Figure 1. Severe disease occurred in 283 of 894 obese patients (31.7%) and 473 of 2111 non-obese patients (22.4%). Among non-obese individuals, mild forms were the most prevalent (41.1%), followed by moderate (36.5%) and severe (22.4%) cases. In contrast, obese patients had a higher frequency of moderate (40.9%) and severe (31.7%) forms, with mild cases accounting for only 27.4% of this group. A Chi-square test was used to assess the association between obesity status and COVID-19 severity. The results indicated a statistically significant difference in the distribution of clinical forms between the two groups (*p* < 0.001), suggesting that obesity is associated with an increased clinical severity of COVID-19 infection. The relative risk (RR) for severe COVID-19 in obese compared to non-obese patients was 1.41 (95% CI 1.25–1.60).

### 3.3. Pulmonary Involvement According to Obesity Status

**Figure 2:**
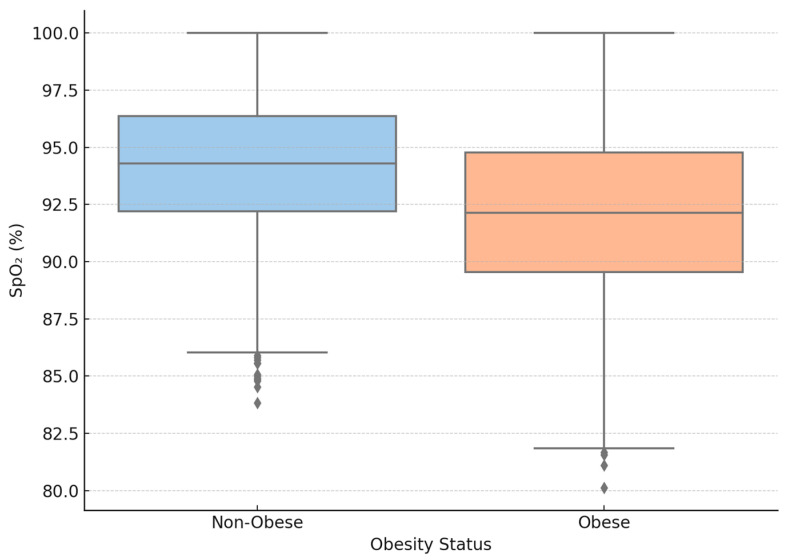
Admission oxygen saturation (SpO_2_) in COVID-19 patients, stratified by obesity status. The boxplot shows a significantly lower median SpO_2_ in obese individuals compared to non-obese patients (*p* < 0.0001, Mann–Whitney U test).

Pulmonary impairment differed significantly between obese and non-obese patients with COVID-19, as illustrated in Figure 2. Mild pulmonary damage (<25%) was more prevalent among non-obese individuals (43.1%) compared to obese patients (33.0%), while severe pulmonary involvement (>50%) occurred more frequently in the obese group (27.9% vs. 22.0%). These differences were statistically significant (*p* < 0.001, the Chi-square test). To further characterize respiratory compromise, oxygen saturation (SpO_2_) at admission was analyzed. The mean SpO_2_ was significantly lower in obese patients (92.1 ± 4.0%) than in non-obese individuals (94.2 ± 3.2%) (*p* < 0.0001, the Mann–Whitney U test). Hypoxemia (defined as SpO_2_ < 90%) was more frequent in the obese group (22.5%) than in the non-obese group (13.7%) (*p* < 0.001, the Chi-square test). The relative risk (RR) for >50% pulmonary involvement in obese compared to non-obese patients was 1.27 (95% CI 1.11–1.45).

When stratifying pulmonary involvement by oxygenation status, 28.0% of obese patients with SpO_2_ ≥ 90% and 27.4% of those with SpO_2_ < 90% had severe lung damage. In comparison, these rates were 21.8% and 24.2% among non-obese individuals, respectively (*p* = 0.006, the Chi-square test). These findings suggest that obesity is associated with increased pulmonary involvement, regardless of baseline oxygen saturation.

### 3.4. Clinical Course During Hospitalization

To assess risk factors for in-hospital mortality, we performed multivariate logistic regression analyses in both the total cohort and separately in obese (Table 2) and non-obese (Table 3) subgroups. In the general population (*n* = 3005), age over 65 years was independently associated with increased risk of death (OR = 1.38, 95% CI: 1.17–1.63, *p* < 0.001), while obesity, low oxygen saturation (SpO_2_ < 90%), male sex, diabetes, hypertension, and chronic pulmonary disease were not significant predictors. All multivariable models were adjusted for vaccination status alongside age, sex, and comorbidities. Overall, there were 165 deaths (18.5%) among obese patients and 571 deaths (27.1%) among non-obese patients out of a total of 736 deaths in the study cohort.

To further explore differential risk patterns, we analyzed the obese and non-obese populations separately. All multivariable analyses were adjusted for vaccination status in addition to age, sex, and comorbidities. Among obese patients (Table 2) (*n* = 894), age > 65 and hypertension were the strongest predictors of in-hospital mortality, though they did not reach conventional significance (OR = 1.32, *p* = 0.083; OR = 1.36, *p* = 0.056, respectively). Interestingly, oxygen saturation <90% had no significant impact in this subgroup (OR = 1.07, *p* = 0.706), suggesting a possible adaptive threshold effect in obese individuals.

In contrast, among non-obese patients (Table 3) (*n* = 2111), age > 65 emerged as a significant independent risk factor for death (OR = 1.41, 95% CI: 1.15–1.72, *p* < 0.001). None of the other variables were significantly associated with mortality in this subgroup either, although SpO_2_ < 90% showed a moderate trend (OR = 1.31, *p* = 0.119).

These findings suggest that while obesity may influence baseline respiratory parameters, age remains the dominant predictor of in-hospital mortality regardless of the body mass index.

To increase statistical power and assess the independent effect of obesity on in-hospital mortality, we also performed a multivariable analysis in the entire cohort (*n* = 3005), including obesity as a covariate alongside age, sex, vaccination status, comorbidities, and baseline oxygen saturation. The results of this model are presented in Table 4. In this model, obesity was not independently associated with mortality after adjustment for all covariates, while age > 65 years and SpO_2_ <90% at admission remained the strongest predictors.

**Figure 3:**
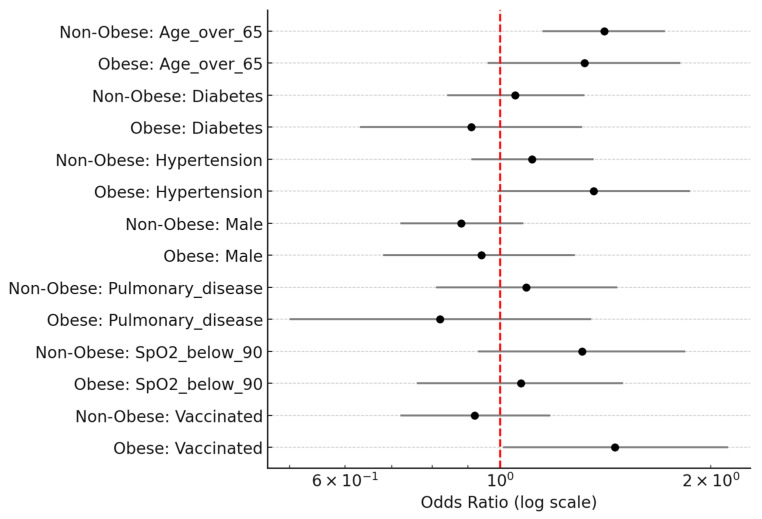
A forest plot illustrating odds ratios (ORs) and 95% confidence intervals for predictors of in-hospital mortality among obese and non-obese patients with COVID-19. Multivariate logistic regression models were fitted separately in each subgroup. While age over 65 years remained a consistent risk factor in both groups, hypertension showed a notable association in obese patients. Odds ratios are plotted on a logarithmic scale; the red dashed line indicates a null value (OR = 1.0).

The impact of individual risk factors on mortality was further visualized in a comparative forest plot (Figure 3), which displays the adjusted odds ratios and confidence intervals for each predictor across both obese and non-obese subgroups. This side-by-side representation highlights the consistent effect of age across groups and the potential differential contribution of hypertension in obese patients. The visual format allows for a direct comparison of the magnitude and direction of associations.

### 3.5. Multivariate Analysis: Predictors of Severe COVID-19

**Figure 4:**
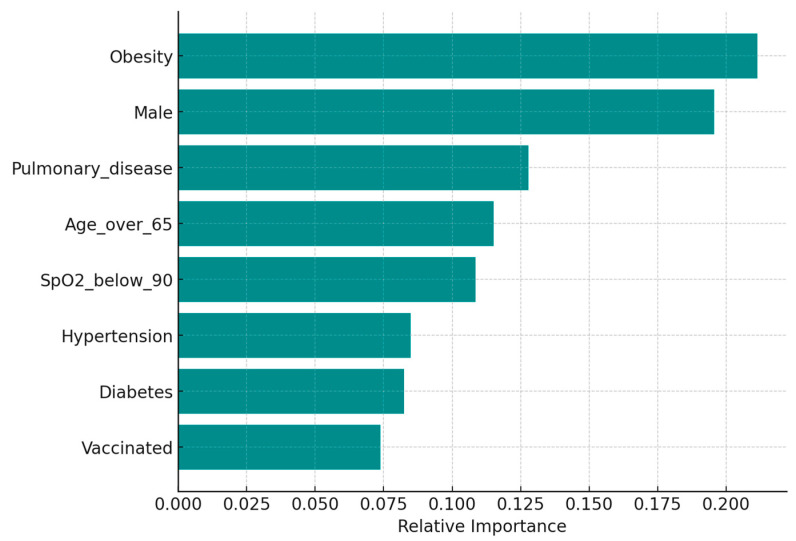
Relative feature importance derived from the gradient boosting model predicting severe COVID-19. Obesity emerged as the most influential predictor, followed by diabetes and oxygen saturation below 90%. Importance values reflect the average contribution of each variable to the decision process within the ensemble model. The model included the following variables: obesity, sex (male), chronic pulmonary disease, age > 65 years, oxygen saturation < 90% at admission (SpO_2__below_90), hypertension, diabetes, and vaccination status. Importance values were calculated as the average gain across all splits where the variable was used in the decision trees, reflecting its overall contribution to model accuracy. Higher values indicate a greater influence on the prediction of severe disease; for example, obesity and male sex were the strongest predictors, followed by chronic pulmonary disease and age > 65 years, whereas vaccination status had the lowest relative contribution.

To better understand the contribution of each predictor in identifying patients at risk for severe COVID-19, we examined feature importance derived from the gradient boosting model. As shown in Figure 4, obesity was the dominant predictor, followed by male sex, chronic pulmonary disease, and age > 65 years. SpO_2_ < 90% at admission, hypertension, diabetes, and vaccination status contributed less to the model’s predictive performance. Feature importance in this context quantifies the relative contribution of each variable to the model’s accuracy; variables with higher scores had a stronger impact on classification decisions, while those with lower scores had minimal influence. These results reinforce the multivariable findings and suggest that obesity has a strong and independent role in determining disease severity.

Taken together, these findings emphasize the predictive strength of obesity in the context of COVID-19 severity. While traditional risk factors such as age and oxygen desaturation did not retain statistical significance in multivariate models, obesity consistently emerged as the most robust determinant across both logistic regression and machine learning approaches. The improved performance of the gradient boosting model, combined with the interpretability offered by feature importance analysis, supports the integration of data-driven algorithms into clinical risk stratification strategies. These insights may facilitate the early identification of high-risk individuals and inform personalized treatment pathways in future COVID-19 surges.

## 4. Discussion

This study demonstrates that obesity is a strong and independent predictor of severe clinical outcomes in patients hospitalized with COVID-19. Our analysis, based on a large single-center cohort, highlights the significant associations between obesity, pulmonary involvement, and the overall severity of disease, as well as the differential impact of age and comorbidities on in-hospital mortality. These findings provide new insights into the role of obesity in respiratory viral infections and support the implementation of risk-adapted management strategies. To reduce confounding, vaccination status was explicitly included as a covariate in all multivariable models and as a predictor in the machine learning analyses. This ensured that the estimated effects of obesity and other covariates on severity and mortality were independent of vaccination effects.

Our results confirm and expand upon previous studies that identified obesity as a major risk factor for adverse COVID-19 outcomes [10,11]. Obese patients in our cohort showed significantly higher rates of severe disease and hypoxemia, despite only modest differences in demographic or comorbidity profiles compared to non-obese individuals. This reinforces the hypothesis that obesity may exert independent pathophysiological effects through mechanisms such as altered respiratory mechanics, pro-inflammatory cytokine profiles, and impaired immune regulation [12,13,14].

Notably, while obesity was a strong predictor of disease severity, it did not independently predict in-hospital mortality in the multivariate model. Instead, age >65 years remained the most consistent and statistically significant predictor of death, particularly in the non-obese subgroup. This suggests that while obesity influences acute respiratory compromise, age and frailty continue to drive late adverse outcomes. Similar dissociation between severity and mortality was reported in multicenter analyses from Europe and the United States [15,16]. Adjusting for vaccination status in all multivariable models minimized the risk of confounding and ensured that the associations observed for obesity and other predictors were independent of vaccination effects.

Importantly, subgroup analysis revealed that predictors of in-hospital mortality may differ between obese and non-obese patients. In the obese group, hypertension and age >65 showed trends toward increased mortality risk, while hypoxemia was not a significant driver. This may reflect an adaptive desensitization to oxygen deficits in obese individuals or limitations in the early recognition of clinical deterioration. Such differences underscore the need for tailored risk assessment tools that account for physiological variances across body composition profiles [17].

These findings reinforce the role of nutritional status as both a modifiable risk factor and a potential therapeutic target in the context of COVID-19 and other acute respiratory illnesses. Obesity is not just a marker of excess adiposity but reflects a complex metabolic and inflammatory state influenced by dietary patterns, micronutrient deficiencies, and altered gut microbiota. In this context, poor nutritional quality—particularly diets rich in ultra-processed foods and low in anti-inflammatory nutrients—may exacerbate immunometabolic dysfunction, thereby increasing vulnerability to viral pathogenesis and systemic complications [18]. Integrating personalized nutrition strategies into public health protocols could enhance resilience against severe infections in high-risk populations [19]. Moreover, routine nutritional screening in hospitalized patients with COVID-19 may help identify those with hidden metabolic fragility, guiding interventions aimed at improving not only acute outcomes but also long-term recovery trajectories [20]. Given the overlapping burden of obesity, metabolic syndrome, and chronic degenerative diseases, these insights further support the development of integrated prevention frameworks rooted in sustainable, evidence-based dietary models [21].

Our study has several strengths, including a large, well-characterized cohort, the use of both classical and modern statistical methods, and detailed stratification by obesity. However, we acknowledge limitations such as the retrospective design, single-center scope, and absence of long-term follow-up. Furthermore, despite robust internal validation, our models require external testing before clinical implementation.

This study has several limitations. First, its retrospective, single-center design may limit the generalizability of the findings to other settings and populations. Second, although we adjusted for key demographic and clinical variables, residual confounding cannot be entirely excluded, particularly from unmeasured factors such as socioeconomic status or unrecorded comorbidities. Third, the classification of vaccination status did not account for the time elapsed since the last dose or for the specific vaccine type, which may have influenced the outcomes. Fourth, this study did not include data on SARS-CoV-2 variants, which could have affected disease severity and mortality over the study period. Finally, the absence of long-term follow-up precludes an assessment of post-discharge outcomes and longer-term complications.

Overall, these findings reinforce the importance of considering obesity as a central factor in COVID-19 risk stratification. Future work should focus on refining prognostic models using multi-omics data, validating machine learning pipelines in prospective cohorts, and exploring the translational potential of nutritional and metabolic interventions in COVID-19 and other acute respiratory conditions.

## 5. Conclusions

This study demonstrates that obesity is an independent risk factor for severe COVID-19 and for extensive pulmonary involvement, even after adjusting for vaccination status, age, sex, and baseline oxygen saturation. While obesity did not independently predict in-hospital mortality, its consistent association with greater disease severity and respiratory compromise underscores its prognostic importance. In contrast, age over 65 years and low admission SpO_2_ emerged as the strongest determinants of mortality across the cohort. These findings highlight the need to include obesity in COVID-19 risk stratification models and to integrate nutritional screening, targeted dietary interventions, and vaccination strategies into both acute care and long-term public health planning for high-risk populations.

## Figures and Tables

**Table 1:** Demographic characteristics by obesity status. Values are expressed as absolute counts with percentages for categorical variables and as the mean ± standard deviation for continuous variables. Statistical comparisons between groups were performed using the Chi-square test for categorical variables and the Mann–Whitney U test for continuous variables. A *p*-value < 0.05 was considered statistically significant.

Variable	Non-Obese	Obese	*p*-Value
Male	1074 (50.9%)	437 (48.9%)	0.337
Female	1037 (49.1%)	457 (51.1%)	0.337
Environment: Urban	1270 (60.2%)	508 (56.8%)	0.031
Environment: Rural	769 (36.4%)	339 (37.9%)	0.031
Age (years)	63.51 ± 14.75	62.77 ± 15.57	0.198
Smokers	584 (27.7%)	279 (31.2%)	0.055

**Table 2:** The multivariate logistic regression model identifying predictors of in-hospital mortality among obese patients with COVID-19. The model included age over 65 years, oxygen saturation below 90% at admission, male sex, diabetes, hypertension, chronic pulmonary disease, and vaccination status. Odds ratios (ORs), 95% confidence intervals (CIs), and *p*-values are reported. None of the predictors reached conventional statistical significance in this subgroup. However, age >65 years (*p* = 0.083) and hypertension (*p* = 0.058) showed a non-significant trend toward an increased risk of mortality.

Variable	OR	95% CI Lower	95% CI Upper	*p*-Value
Age > 65	1.32	0.96	1.81	0.083
SpO_2_ < 90	1.07	0.76	1.5	0.706
Male	0.94	0.68	1.28	0.681
Diabetes	0.91	0.63	1.31	0.617
Hypertension	1.36	0.99	1.87	0.056
Pulmonary disease	0.82	0.5	1.35	0.431
Vaccinated	1.46	1.01	2.12	0.045

**Table 3:** The multivariate logistic regression model showing predictors of in-hospital mortality among non-obese patients with COVID-19. The model included age over 65 years, oxygen saturation below 90% at admission, male sex, diabetes, hypertension, chronic pulmonary disease, and vaccination status. Odds ratios (ORs), 95% confidence intervals (CIs), and *p*-values are reported. Age > 65 years was a statistically significant predictor of mortality (*p* < 0.001) in this subgroup. Hypertension showed a non-significant trend toward increased risk (*p* =0.311). The model was adjusted for vaccination status to account for its significant influence on mortality risk and to reduce confounding effects in the analysis of other variables.

Variable	OR	95% CI Lower	95% CI Upper	*p*-Value
Age > 65	1.41	1.15	1.72	< 0.001
SpO_2_ < 90	1.31	0.93	1.84	0.119
Male	0.88	0.72	1.08	0.210
Diabetes	1.05	0.84	1.32	0.648
Hypertension	1.11	0.91	1.36	0.311
Pulmonary disease	1.09	0.81	1.47	0.586
Vaccinated	0.92	0.72	1.18	0.517

**Table 4:** Multivariable logistic regression model for in-hospital mortality in the entire study cohort (*n* = 3005), including obesity and other key covariates. Adjusted odds ratios (ORs) with 95% confidence intervals (CIs) and *p*-values are reported.

Variable	Adjusted OR	95% CI Lower	95% CI Upper	*p*-Value
Age > 65 years	1.45	1.24	1.69	<0.001
Male sex	1.18	1.01	1.38	0.041
Obesity	1.12	0.92	1.36	0.260
Vaccinated	0.88	0.74	1.05	0.160
Diabetes	1.21	1.03	1.43	0.022
Hypertension	1.09	0.93	1.27	0.280
Chronic pulmonary disease	1.15	0.92	1.44	0.220
SpO_2_ < 90% at admission	1.64	1.38	1.95	<0.001

## Data Availability

The data supporting the findings of this study are available upon reasonable request from the corresponding author. Access will be granted in anonymized form and only with approval from the institutional ethics committee, in accordance with data protection regulations.
